# Water-Induced Restructuring of the Surface of a Deep Eutectic Solvent

**DOI:** 10.1021/acs.jpclett.1c03907

**Published:** 2022-01-12

**Authors:** Rahul Gera, Carolyn J. Moll, Aditi Bhattacherjee, Huib J. Bakker

**Affiliations:** AMOLF, Science Park 104, 1098 XG Amsterdam, The Netherlands

## Abstract

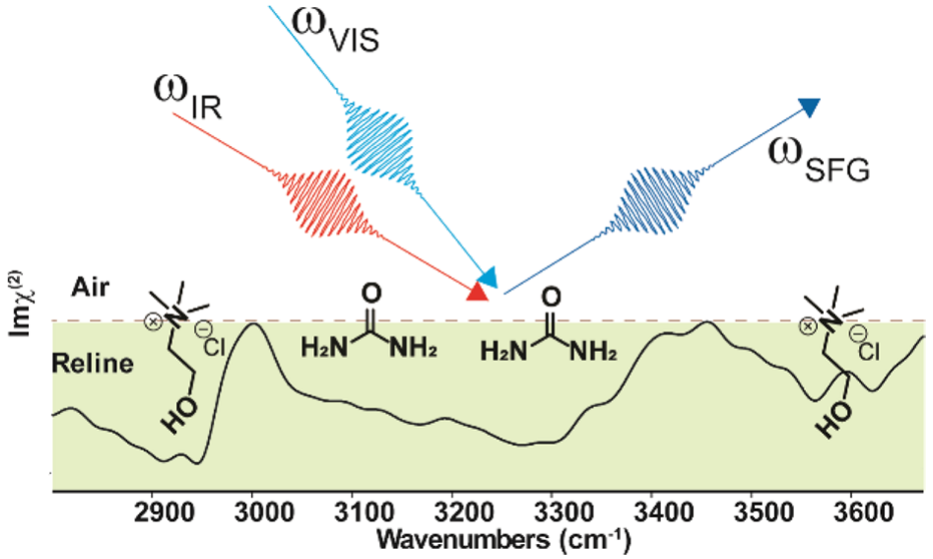

We study the molecular-scale structure of the surface of Reline, a DES made from urea and choline chloride, using heterodyne-detected vibrational sum frequency generation (HD-VSFG). Reline absorbs water when exposed to the ambient atmosphere, and following structure-specific changes at the Reline/air interface is crucial and difficult. For Reline (dry, 0 wt %, w/w, water) we observe vibrational signatures of both urea and choline ions at the surface. Upon increase of the water content, there is a gradual depletion of urea from the surface, an enhanced alignment, and an enrichment of the surface with choline cations, indicating surface speciation of ChCl. Above 40% w/w water content, choline cations abruptly deplete from the surface, as evidenced by the decrease of the vibrational signal of the −CH_2_– groups of choline and the rapid rise of a water signal. Above 60% w/w water content, the surface spectrum of aqueous Reline becomes indistinguishable from that of neat water.

In the ongoing search for environment-friendly solvents, deep eutectic solvents (DESs) are emerging as a promising candidate to replace volatile organic solvents and possibly ionic liquids.^1−6^ DESs are two-component mixtures, generally made up of an organic salt and a hydrogen bond donor in a specific molar ratio, with an eutectic melting point that is much lower than the melting points of the pure constituents.^3−5,7−10^ DESs are nonflammable, have a low vapor pressure and high thermal stability, and can usually be produced at low costs. The unique physicochemical properties of these binary mixtures are attributed to the nature and strength of the intermolecular interactions, predominantly hydrogen bonds.^3−5,7−10^ One of the most compelling arguments for using DESs is that their chemical composition allows them to be used as a green solvent.^3−18^ As a result, DESs have already found applications in the fields of homogeneous bulk-phase chemistry related to organic chemistry reactions and mesoporous material synthesis,^19−21^ electrochemistry,^22,23^ industrial processes^18,24^ such as metal ion sequestration,^25^ and biotransformations.^26−28^

Reline is an archetype DES, made from choline chloride (ChCl) and urea in a molar ratio of 1:2.^9,10,14−18,29,30^ The melting point of Reline (12 °C) is significantly lower than the melting points of ChCl (303 °C) and urea (134 °C). Several experimental and theoretical studies have revealed the presence of specific nanostructures in the mixture.^4,5,7,9,30^ Conductivity, viscosity,^16,31^ and neutron diffraction measurements^9,14,30^ of Reline show a three-dimensional intermolecular H-bonding network with a long-range ordering. In these studies, strong hydrogen bond interactions are observed between the chloride anion (Cl^–^) and urea as well as between the choline cation (Ch^+^) and urea.^9,14,30^

Many DESs including Reline tend to absorb water when exposed to the ambient atmosphere.^32^ Slight modifications in the composition, functional derivatization of components, or doping with a third component lead to variabilities in the melting point and other physical properties of the system.^13−18^ The addition of controlled amounts of water to high-viscosity DESs has emerged as an attractive route to enhance fluidity, solvation, and conductivity.^32^ The modulation of the viscosity of DES by a controlled addition of water has found applications in food processing, enzyme actions, pharmaceuticals, and cosmetics.^33,34^ It is also reported to be beneficial in the quality of electroplating produced from eutectic solvents.^35^ Adding water is an environmentally friendly method to change the properties of a DES. Therefore, it is important to understand how the addition of water affects the structure of DES on the molecular scale, including their surface structure. An interesting and important research question is to what extent water disrupts the nascent hydrogen-bonding structures of a DES. Up to now, this question has mainly been addressed by bulk-specific, structural probes.^16,32^

In this work, we report the molecular structure at the Reline/air interface and the surface structural evolution with increasing water content (0%–70% w/w or 0–92 mol %; see Table S1 in the Supporting Information). Knowledge of the molecular structure at the air–liquid interface of DES is expected to be crucial in surface-selective applications such as molecular extractions and heterogeneous catalysis.^4^ We utilize the vibrational frequencies of urea, choline chloride, and water to identify changes in the molecular structures at the interface. With increasing hydration, we find a stepwise depletion of urea and ChCl from the surface which has important implications for molecular separation and catalysis.

Figure 1 shows the FTIR spectrum of pure Reline (black) and different weight percentages of water up to 70% w/w. The spectrum of pure Reline agrees with the previously reported infrared spectrum of Reline.^7,29^ The broad infrared band centered at ∼3250 cm^–1^ has multiple peaks due to the (i) asymmetric (ν_N–H_^as^, at ∼3400 cm^–1^) and symmetric stretches (ν_N–H_^ss^, at ∼3310 cm^–1^) of −NH_2_ in urea, (ii) overtone modes of the carbonyl (⟩C=O) stretch and −NH_2_ bending modes involved in a Fermi resonance with the N–H vibrations of urea (ν_N–H_^Fr^, at ∼3185 cm^–1^), and (iii) O–H stretch vibration from Ch^+^ hydrogen-bonded to Cl^–^ at ∼3255 cm^–1^.^7,30^ The weaker features observed at 3000 cm^–1^ in the trailing red edge of the broad IR absorption band are due to CH stretching normal modes of the methyl (−CH_3_) and methylene (−CH_2_−) groups in ChCl (Figure S1). Reline also shows two vibrational bands at ∼1600 and ∼1660 cm^–1^ (Figure 1) assigned to mixed modes of −NH_2_ bending and ⟩C=O stretch vibrations of urea.^36,37^ The ∼1660 cm^–1^ band has a major contribution from the ⟩C=O mode, while the mode at ∼1600 cm^–1^ has a major contribution from the −NH_2_ bending mode.^7,30^ A broad feature observed at ∼1725 cm^–1^ (see Figure 1) has been previously assigned to non-hydrogen-bonded urea in Reline.^7^

**Figure 1:**
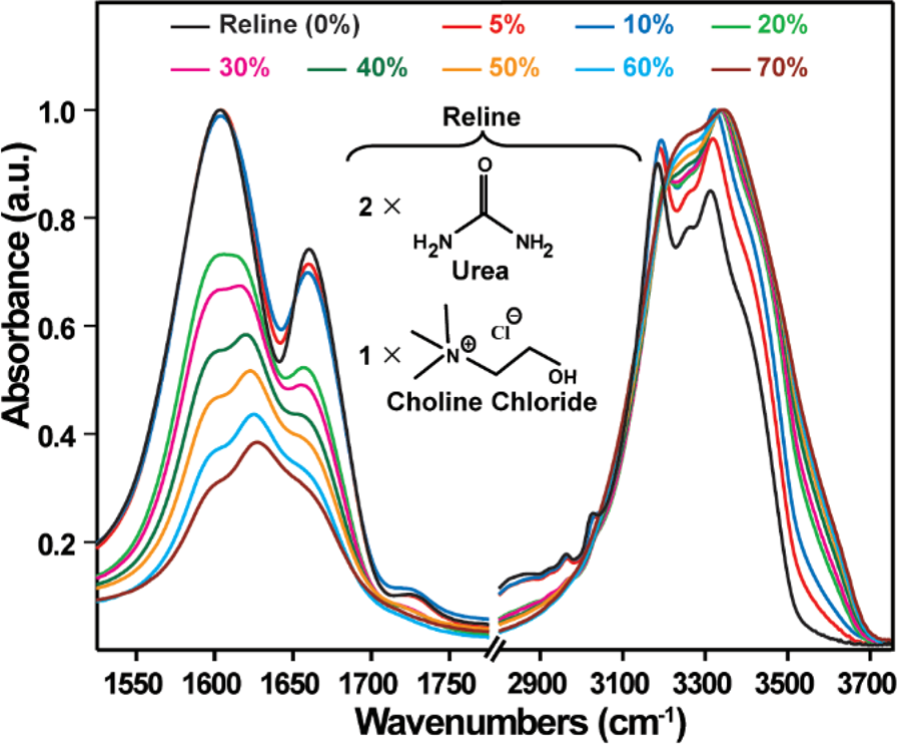
FTIR spectrum of Reline acquired in ATR mode. Spectrum of Reline with increasing increment of water addition by weight percent (w/w %).

Increasing the hydration of Reline leads to a loss of spectral structure due to a broadening of the infrared bands in the ∼3100–3500 cm^–1^ frequency region (multicolored traces in Figure 1). This loss of structure can be explained by the increasing contribution of the O–H stretch vibrations of water to the signal. Similar changes are observed in the frequency region of the ⟩C=O stretch and −NH_2_ bending vibrations of urea. With increasing water content, the spectra reveal an increasing contribution of the water bending mode at ∼1630 cm^–1^, while the features of H-bonded urea at ∼1600 and ∼1660 cm^–1^ in Reline decrease.

To achieve insight into the structure and orientation of ChCl, urea, and water at the surface of Reline–water mixtures, we performed HD-VSFG measurements (Figure 2).^38−40^ The frequency range of the mid-IR beam is ∼2800 to ∼3700 cm^–1^. It covers the C–H modes of Ch^+^ (region I), N–H stretching modes of urea (region II), and O–H of ChCl (region III) (also see the |χ^(2)^|^2^ spectrum in Figure S2 and raw data in Figure S5 collected by using the HD-VSFG measurement in the Supporting Information). This division into different frequency regions is made to facilitate the discussion of the major spectral changes. The Im χ^(2)^ spectrum of Reline surface (black trace, Figure 2) is quite different from the bulk FTIR spectrum (Figure 1). In the frequency region of ∼2800 to ∼3000 cm^–1^ (region I) we attribute the broad feature to the C–H stretch vibrations of the alkyl groups of Ch^+^. The main contributions are from the methylene group (−CH_2_−) of the alkyl chain and the methyl group (−CH_3_) at the ammonium headgroup of Ch^+^.^41−49^ The broad and unstructured nature of the CH vibrations in region I indicates the presence of significant structural heterogeneity at the Reline/air interface. The Im χ^(2)^ spectrum of Reline shows a negative sign for the vibrational features in region I, which implies that the −CH_3_ group of the ammonium headgroup of Ch^+^ points into the air.^50−54^ For pure Reline we observe a strong signal at ∼2940 cm^–1^, which is assigned to the asymmetric C–H stretch vibration from the methylene (−CH_2_−) groups of the hydrophobic tail of Ch^+^ (see Figure 1 for Ch^+^ structure).^41,47,55,56^ The feature at ∼2940 cm^–1^ indicates the presence of a significant gauche conformation of Ch^+^ at the surface, indicative of structural heterogeneity.^56^

**Figure 2:**
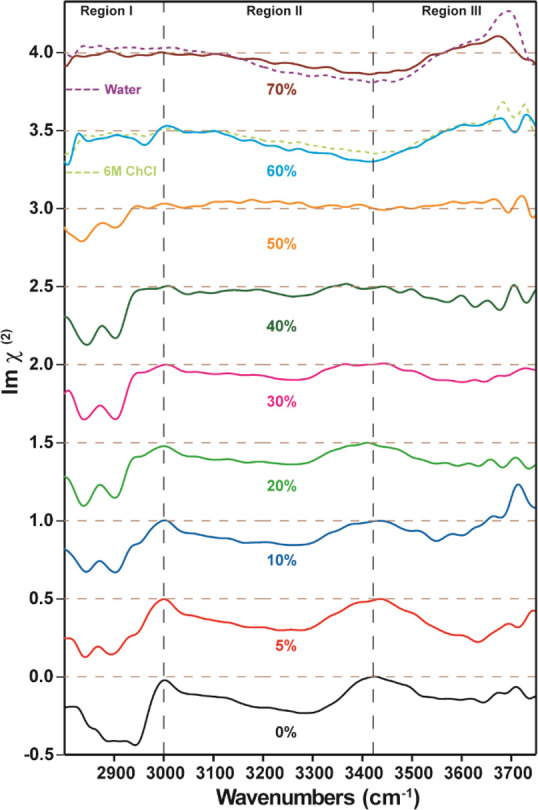
Im χ^(2)^ spectra of Reline with increasing addition of water by weight percentage from 0% to 70% measured by using HD-VSFG. Region I contains the CH stretch vibrations of ChCl, region II contains the N–H stretch vibrations from urea, and region III contains the O–H stretch vibrations from ChCl. The spectrum at 60% w/w is compared with the spectrum of a 6 M ChCl solution (dashed light green line) because the concentration of ChCl in a Reline sample at 60% w/w is ∼6 M. The spectrum at 70% w/w is compared to that of neat water (dashed purple line). All spectra are plotted with a vertical offset for clarity. The brown dashed lines represent the zero lines of the spectra. The HD-VSFG spectrum is normalized by dividing the spectrum by the signal from a nonresonant reference (z-cut quartz); this inevitably give rise to noise in regions where IR intensity is low (also see Figure S5).

In region II spanning from ∼3000 to ∼3400 cm^–1^, we observe a broad response containing vibrational features from urea corresponding to ν_N–H_^Fr^ and ν_N–H_^ss^.^7,57,58^ The sign of the Im χ^(2)^ is negative with a maximum at ∼3300 cm^–1^. The spectrum in this region has an asymmetric shape that is characteristic of N–H stretch vibrations.^53,57^ The negative sign of the Im χ^(2)^ shows that the N–H groups of urea are pointing downward into the bulk, away from air.^53,58^ In addition, a broad band is observed at frequencies >3400 cm^–1^ in region III, which is assigned to the stretching vibration of the weakly hydrogen-bonded O–H group of Ch^+^.^48,59,60^ For comparison, Figure 2 also shows the HD-VSFG spectrum of the neat water/air surface (dashed purple trace) which is similar to the spectrum observed in previous HD-VSFG measurements of water.^61−64^ The broad feature centered at ∼3400 cm^–1^ is assigned to the OH-stretch vibrations of hydrogen-bonded water molecules, while the sharp feature ∼3700 cm^–1^ is assigned to non-hydrogen-bonded, dangling OH groups sticking out of the surface.^38,39^

As illustrated in Figure 2, adding water to Reline leads to significant changes in the Im χ^(2)^ spectrum. The broad features of the C–H stretches in region I are more structured than for pure Reline, revealing two distinguishable features at ∼2830 and ∼2900 cm^–1^. These features are assigned to the symmetric C–H stretch vibration from the methyl (−CH_3_) group of Ch^+^.^41,47,48,65^ The spectral contribution from the −CH_2_– groups along the chain is expected to be weak, as their contributions largely cancel each other because of their antisymmetric positioning with respect to the alkyl chain.^56^ The narrowing of the broad unstructured region into two sharp narrow features in region I indicates that the surface structure of Ch^+^ becomes more uniform and ordered when the water content is increased. This type of structural change is commonly observed for surfactants at water surfaces when the concentration of polar species is increased.^56,66−69^ With increasing water content, the Ch^+^ ions acquire a more ordered and uniform alignment at the surface, thus removing the gauche conformation, as evident from the decrease of the signal at ∼2940 cm^–1^.^56^ The spectral features in region II also change upon an increase of the water concentration, where the contribution to the vibrational spectrum from urea decreases (see Figure 2 and Figure S2). These observations indicate that the urea concentration at the surface decreases with increasing water content. The broad feature at frequencies >3400 cm^–1^ in region III, assigned to the hydrogen-bonded O–H stretch vibration of Ch^+^, is largely unaffected by the presence of water up to 30% w/w (or ∼67 mol %).^48,59^

Increasing the water concentration beyond 40% w/w (or ∼76 mol %) leads to a significant change of the Im χ^(2)^ spectrum, as seen in Figure 2. At 50% w/w (or ∼83 mol %) concentration of water, the contribution of the N–H stretch vibrations of urea becomes negligible. The contribution from the C–H stretch vibrations also decreases significantly (also see Figure S2). At the same time, a broad vibrational band appears between ∼3000 and ∼3600 cm^–1^ which is characteristic of the OH stretch vibrations of hydrogen-bonded water molecules. At 60% w/w water content (or ∼88 mol %) the surface is mainly occupied by water; further, the OH response of non-hydrogen-bonded water is seen at ∼3700 cm^–1^. The spectrum obtained in a control measurement of the HD-VSFG spectrum of an aqueous solution of 6 M ChCl matches well with the spectrum of Reline with 60% w/w water (see Figure 2, green dotted line trace for ∼6 M ChCl), showing that urea does not contribute to the surface signal at this level of hydration.^70^ In contrast, a Reline sample with 70% w/w (or ∼92 mol %) has a very similar spectrum as that of pure water (see Figure 2, purple dotted line trace), showing that at these water concentrations neither urea nor choline contributes to the surface-specific signal.^70^ The surface spectrum thus becomes indistinguishable from that of neat water.

Figure 3 shows the HD-VSFG measurements of Reline and Reline–water mixtures in the spectral region of 1500–1800 cm^–1^. As noted in Figure 1, this region contains the vibrational modes of urea (⟩C=O stretch and −NH_2_ bend) and that of the water bending mode.^7,30,71−73^ The experimental details of the setup and the measurement procedure are presented in the Supporting Information. The HD-VSFG spectrum in the 1500–1800 cm^–1^ region shows two vibrational bands centered at ∼1590 and ∼1660 cm^–1^, similar to the FTIR spectrum.^36,37^ The Im χ^(2)^ values of the two bands have a negative sign which implies that the ⟩C=O group is oriented with its oxygen toward the air (i.e., oxygen atom further away from the bulk than the carbon atom).^53^ This result agrees with the observation that the −NH_2_ groups of urea are oriented toward the bulk, as supported by the Im χ^(2)^ spectra of Figure 2.

**Figure 3:**
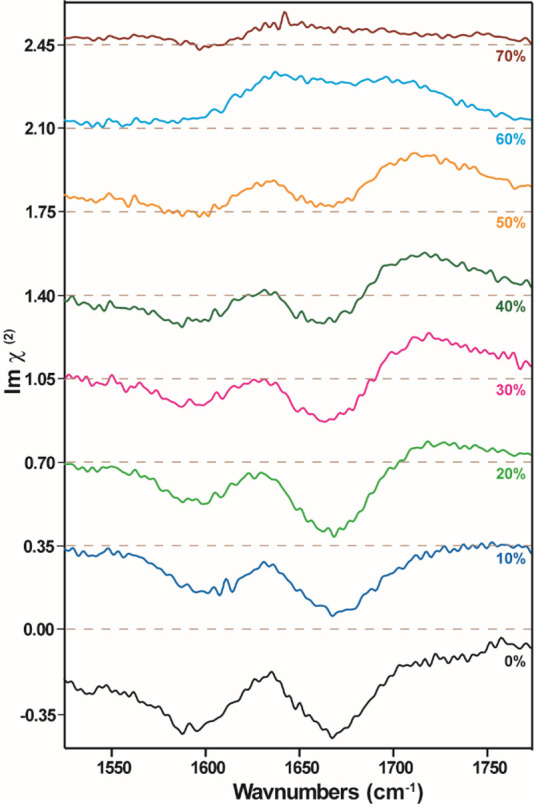
Im χ^(2)^ spectrum of Reline with increasing addition of water by weight percentage from 0% to 70% obtained with HD-VSFG. All the spectra plotted for samples from Reline with 10% w/w water onward are offset by 0.35 au on the *y*-axis. The brown dashed lines represent the zero line for each spectrum.

We find that the bands at ∼1590 and ∼1660 cm^–1^ decrease in amplitude when the water content is increased, similar to what we observed in region II of Figure 2 (also Figures S2 and S3). This observation confirms that urea gets progressively depleted from the surface with increasing hydration and that its signal completely vanishes for mixtures containing >50% w/w (∼83 mol %) water. The C–H features continue to be prominent at these concentrations of water (Figure 2), which is indicative of water-induced speciation of Ch^+^ at the surface. For Reline an additional feature at ∼1720 cm^–1^ is observed when the water content increases above 20% w/w. This band can be assigned to the ⟩C=O stretch vibration of non-hydrogen-bonded urea.^7^ At ∼ 60% w/w (or ∼88 mol %) water concentration the bands at ∼1590 and ∼1660 cm^–1^ of urea disappear, and the measured response is due to non-hydrogen-bonded urea at ∼1720 cm^–1^ and the water bending mode which appears as a positive peak at ∼1650 cm^–1^.^63,73,74^ To support the above assignment for urea in this region, we also performed measurements on Reline containing deuterated urea and adding heavy water (D_2_O). The −ND_2_ deformation vibration of deuterated urea absorbs at much lower frequency region of ∼1200 cm^–1^,^30^ and thus the two bands of mixed character at ∼1590 and ∼1660 cm^–1^ of urea are replaced by a single band at ∼1635 cm^–1^ of the ⟩C=O stretch vibration of deuterated urea (see Figure S4).^30,36^ The feature of non-hydrogen-bonded urea is now observed at ∼1705 cm^–1^, confirming our assignment.

To illustrate the structural changes taking place at the surface upon the addition of water in more detail, Figure 4 shows the absolute amplitude of Im χ^(2)^ as a function of water content at three selected frequencies of 2902, 3050, and 3425 cm^–1^, representing the signals of choline, urea, and water, respectively (the amplitudes are obtained by performing five-point adjacent averaging, spanning a spectral width of ∼3 cm^–1^). At these frequencies, there is minimal overlap with contributions from the other constituents. It is clearly seen that the drop in the amplitude of the response of urea is much more gradual than that of Ch^+^ (of ∼2902 cm^–1^). The rise in the signal of water at the surface is quite abrupt, occurring at a water concentration of ∼50% w/w (or ∼83 mol %).

**Figure 4:**
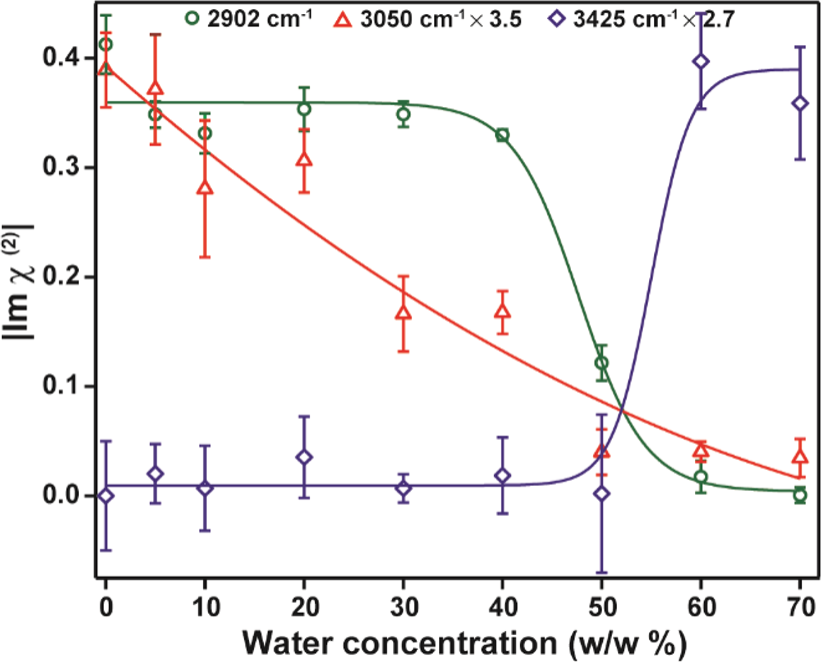
Absolute Im χ^(2)^ amplitude at three different frequencies for water–Reline mixtures as a function of the water concentration. The solid lines are guides to the eye. The amplitudes of the Im χ^(2)^ of urea and water are multiplied by a factor of 3.5 and 2.7, respectively, to enable a better comparison with the changes observed for choline.

From the above observations, the following molecular picture for the reorganization of the liquid arises. At the interface of neat Reline, both ChCl and urea are present. When small amounts of water are added, this water is absorbed in the bulk of the liquid, as evident from the fact that no surface-specific water features are observed. This finding agrees with previous bulk-specific, structural probes of Reline–water mixtures, which showed that choline-based DESs are capable of retaining their nascent H-bonded structures up to ∼40 wt % of water, where water molecules get sequestered into nanostructured domains around the choline cations (Ch^+^).^14,16,32^ Our results thus confirm that up to 40% w/w (or ∼76 mol %) of water content, the added water is absorbed into small clusters in the bulk of the DES.

Our results also show that adding water induces a change of the ratio of urea and Ch^+^ at the surface, in favor of the latter species. We also observe that the choline ions become increasingly well ordered. Adding water thus, interestingly, leads to a DES of a different composition at the surface than in the bulk, which could have correspondingly different physicochemical properties (e.g., melting point).^16,32,75,76^

From 50% w/w (or ∼83 mol %) water content onward, we observe the rise of vibrational features from water at the surface. At this water content, choline gets depleted from the surface and is replaced by water. Above 60% w/w (or ∼88 mol %) water content, the surface of the Reline–water mixture becomes indistinguishable from that of pure liquid water. Previous bulk studies showed that at ∼50 wt % of water the DES molecular structures get significantly disrupted and that at even higher water fractions DES–water mixtures appear to behave like aqueous solutions of the individual components.^14^ Combining these previous results with our findings, we conclude that at water concentrations >50% both urea and Ch^+^ become well solvated by water in the bulk of the mixture, which implies that the DES-specific hydrogen-bond structures are completely disrupted. At these water concentrations, the mixture behaves like an aqueous solution of the separate components urea and Ch^+^, showing a dominant response of water at its surface.

We study the molecular properties at the surface of the deep eutectic solvent Reline (ChCl:urea = 1:2) with intensity and heterodyne-detected sum frequency generation (HD-VSFG) spectroscopy. In particular, we report the effect of adding water to Reline. For pure Reline (no water added), we find that both urea and choline are observed at the surface. The HD-VSFG measurements show that the N–H groups of urea have a net orientation toward the bulk, and the ⟩C=O group has a net orientation toward the air. For O–H, N–H, and C–H stretch vibrations, the modulation of the dipole moment and the polarizability induced by the vibration have the same dependence on the phase of the vibration. In this case Im(χ^(2)^) is positive if the positive charge of the dipole is closer to the surface, and Im(χ^(2)^) is negative if the positive charge of the dipole is closer to the bulk. For the N–H group of urea, the hydrogen is positively charged with respect to the N atom, and the negative sign of Im(χ^(2)^) thus shows that the N–H group is pointing downward with its H atom pointing toward the bulk. For the ⟩C=O group, the carbon atom is more positively charged, and a negative Im(χ^(2)^) implies that the (positive) carbon atom is closer to the bulk, which means that the ⟩C=O group points with its oxygen atom toward the surface. These measurements also show that the CH_3_ group of the ammonium headgroup of Ch^+^ has a net orientation toward the air, i.e., away from the bulk, and that the distribution of the choline ions (Ch^+^) is quite heterogeneous.

Adding water to Reline leads to a gradual depletion of urea from the surface and a better aligned and less heterogeneous distribution of Ch^+^ ions at the air interface. The effect of water is that it creates a DES of a different composition at the surface than in the bulk. At water concentrations above 40% w/w (or ∼76 mol %), the surface undergoes an abrupt reorganization: the choline ions are displaced into the bulk of the solution, and water molecules accumulate at the surface. Above 60% w/w (or ∼88 mol %) water content, the VSFG spectrum becomes highly similar to that of pure liquid water.

We find that doping of a DES with a third component, e.g., water, can modify its surface properties and may be used in surface-related applications such as selective extraction of chemical reagents. Future surface-specific experimental investigations and theoretical modeling of different DESs will expand the use of these highly promising solvents in the fields of heterogeneous catalysis, biocatalysis, and molecular extraction.

## Experimental Methods

### Materials

Choline chloride (purity ≥98%), urea (purity ≥99%), urea-*d*_4_ (purity ≥98%), and deuterium oxide (purity ≥99%) were purchased from Sigma-Aldrich.

### Preparation of Reline

The preparation of the Reline samples is based on previously reported protocols.^17,75^ Choline chloride is dried in an oven which is set at 80 °C. The complete removal of water content from the hygroscopic crystals of ChCl is verified by ATR-FTIR spectroscopy performed by using a Bruker Vertex80v FTIR-ATR spectrometer. Urea is used without further purification. To make the DES Reline, we mix the dried choline chloride and urea in a 1:2 molar ratio in a 25 mL sample vial. The solid mixture is stirred by using a magnetic stirrer and simultaneously heated to 60–70 °C under a dry nitrogen atmosphere until it forms a homogeneous and colorless liquid mixture.^17^ The mixture (Reline) is subsequently cooled to room temperature and stored in a sample vial sealed with parafilm. To remove any water contamination introduced during sample storage, the FTIR and SFG experiments were performed after heating the samples to 70 °C under nitrogen purge with constant stirring for at least 1 h.^75^ The absence of water in the resulting sample is verified by FTIR measurements that do not show any observable spectral signatures of water in the stretching or bending regions.

### FTIR Measurements

We performed Fourier transform infrared (FTIR) absorption measurements of the samples in attenuated total reflection (ATR) mode using a Bruker Vertex80v spectrometer equipped with an ATR module (Platinum ATR Diamond). The layer thickness that is probed in the ATR geometry is typically on the order of a few micrometers, determined by the decay length of the evanescent field, which is a function of wavelength, angle of incidence, and the refractive indices of the ATR crystal and the sample.^77^ The spectral resolution of the ATR spectra is 2 cm^–1^.

### HD-VSFG Measurements

Details of the experimental setup have been discussed in previous publications and are presented the Supporting Information.^58,68,78−80^ Briefly, VSFG is performed by focusing two laser pulses and overlapping them on the sample spatially and temporally. One of the laser pulses, ω_IR_, is from a mid-infrared (mid-IR) source and is resonant with the vibrational frequencies of the molecules present at the surface. The interaction with the second laser pulse, ω_VIS_ (∼800 nm), creates a third beam at the sum frequency, ω_SFG_ (ω_SFG_ = ω_IR_ + ω_VIS_), which is detected with a CCD camera. The sum-frequency intensity is enhanced at frequencies for which the corresponding infrared frequency is resonant with a molecular vibration at the surface. This frequency dependence is expressed in the second-order nonlinear susceptibility, χ^(2)^(ω). As an extension of conventional VSFG, heterodyne-detected sum frequency generation (HD-VSFG) provides both the real and imaginary parts of the second-order nonlinear susceptibility, χ^(2)^. Im χ^(2)^(ω) represents the vibrational spectrum of the molecules at the interface, while the FTIR/ATR spectrum represents the vibrational spectrum of the bulk.^62,64,81^ All measurements are performed by using an ssp-polarization configuration for the three beams involved (polarizations of ω_SFG_, ω_VIS_, and ω_IR_, respectively). In this configuration, the sign of Im χ^(2)^ contains information about the orientation of the molecular group that carries the normal vibrational mode along the axis perpendicular to the surface.^38−40,62,64,81^ The HD-VSFG spectra of Reline with different water concentrations are measured in two series. The first series was recorded for water concentrations 0%–40% w/w, and the second series was recorded for water concentrations 40%–70% w/w.

## Supporting Information Available

Additional HD-VSFG data and experimental method information (PDF)
