# Supplementary material for: Water-Induced Restructuring of the Surface of a Deep Eutectic Solvent
Source: J Phys Chem Lett. 2022 Jan 12;13(2):634–41. doi: 10.1021/acs.jpclett.1c03907 (PMC8785180; doi:10.1021/acs.jpclett.1c03907)
Supplement: Supplementary file 1 — jz1c03907_si_001.pdf [file jz1c03907_si_001.pdf]

Supporting Information

Water-induced Restructuring of the Surface of a Deep Eutectic Solvent.

Rahul Gera*, Carolyn J. Moll, Aditi Bhattacherjee*[†], Huib J. Bakker.

AMOLF, Science Park 104, 1098 XG Amsterdam, The Netherlands.

VIBRATIONAL SUM-FREQUENCY GENERATION

Vibrational sum-frequency generation (VSFG) is a surface specific spectroscopic technique that has significantly advanced over the last decades. VSFG is carried out by focusing two laser pulses ω_{IR}, ω_{VIS}, and overlapping them on the sample spatially and temporally. The ω_{IR}, mid-infrared pulse, is resonant with vibrational frequencies of molecules under investigation present at the interface. ω_{VIS} (~800 nm) is the frequency of a visible pulse used for up-converting the signal to visible region for detection on a CCD camera. The overlap of ω_{IR} and ω_{VIS} on the sample results into the generation of a third beam, ω_{SFG}, the sum frequency ($\omega_{SFG} = \omega_{IR} + \omega_{IR}$). Within the dipole approximation light at ω_{SFG} can only be generated in a non-centrosymmetric medium, so for most systems light at ω_{SFG} is only created at the surface, where the symmetry is broken, thus making the process highly interface specific.

The intensity of the sum frequency (I_{SFG}) light $I_{SFG} \propto |\chi^{(2)}|^2 I_{VIS} I_{IR} L_{SFG}^2 L_{IR}^2 L_{VIS}^2$, where $\chi^{(2)}$ is the second-order nonlinear susceptibility, I_{VIS}, I_{IR} are the intensities of the incoming visible and mid-infrared radiation, respectively, and L_{SFG}, L_{IR}, L_{VIS} are the Fresnel coefficients for the SFG, IR and the visible beams, respectively.[1,2] We also measure the VSFG spectrum of a reference sample for which the sum-frequency generation is non-resonant, to normalize the measured signal on the spectral intensity distribution of the infrared pulse. $\chi^{(2)}_{ref}$ is independent of ω_{IR} and ω_{VIS}.[1,2] Thus using,

$$\frac{I_{SFG}(\omega_{SFG})}{I_{ref}(\omega_{SFG})} = \frac{|\chi^{(2)}\,(\omega_{SFG} = \omega_{IR} + \omega_{VIS})|^2}{|\chi^{(2)}_{ref}\,(\omega_{SFG} = \omega_{IR} + \omega_{VIS})|^2}$$

a frequency-dependent |χ(2)|2 or an Intensity VSFG spectrum is obtained and can often be expressed as a sum of Lorentzian vibrational resonances and a non-resonant background signal, i.e.,

$$\chi^{(2)}(\omega_{SFG} = \omega_{IR} + \omega_{VIS}) = A_{NR} + \sum_n \frac{A_n}{\omega_n - \omega_{IR} - i\Gamma_n}$$

where, ω_n, A_n, and Γ_n are the frequency, amplitude, damping constant of the vibrational mode n of the molecule at the interface respectively, and A_{NR} is the non-resonant background. A direct determination of $\chi^{(2)}(\omega_{SFG})$ from the conventional intensity VSFG is not possible as I_{SFG} is proportional to $|\chi^{(2)}|^2$, which strongly depends on the interference of the non-resonant background and resonant terms coming from molecular vibrations. To disentangle the contribution from the non-resonant background and to obtain the real and imaginary part of $\chi^{(2)}$, the VSFG technique has been developed to heterodyne detected VSFG (HD-VSFG), providing direct information on the real and imaginary parts of $\chi^{(2)}$.

EXPERIMENTAL SETUP:

Here we give a brief description of our implementation of HD-VSFG. A more detailed description of the setup can be found in previous works.[3,4]

For the measurements performed in the region of ~2800-~3700 cm^{-1} region, we used ~3 mJ, 35 fs pulses from a regenerative Ti:sapphire amplifier (Coherent) working at 1 kHz repetition rate centered at 800 nm are split into two parts. About 2 mJ of the fundamental beam is used to generate a mid-infrared (mid-IR) beam using a tunable home-built optical parametric amplifier (OPA) and a difference-frequency generation (DFG) stage. The ω_{IR} from the OPA + DFG was centered at ~3000 nm and the pulses had an energy of ~10-12 μJ. Another part of the fundamental beam is sent through an etalon to stretch the femtosecond beam to a few picoseconds and to narrow the frequency bandwidth to 15 cm^{-1}. This beam acts as ω_{Vis}. The two beams from the OPA (ω_{IR}) and the etalon (ω_{Vis}) are focused onto the sample in spatial and temporal overlap to generate light at the sum frequency (ω_{SFG}). In HD-VSFG we generate ω_{SFG} from both a gold substrate that acts as a local oscillator (LO), and from the sample. The two SFG responses are delayed in time with respect to each other by passing the local oscillator ω_{SFG} beam through a silica plate (~1 mm). The two ω_{SFG} beams are sent into a monochromator and detected with an electron-multiplied charge-coupled device (EMCCD, Andor Technologies). The interference pattern of the two beams makes it possible to extract the real and imaginary part of $\chi^{(2)}$ using Fourier transformation. Im$\chi^{(2)}$ is obtained by comparing the HD-VSFG signal with a reference HD-VSFG signal of which the phase of the SFG light is known. We obtain the reference HD-VSFG by replacing the sample by a z-cut quartz crystal. The typical acquisition time of an HD-VSFG spectrum is 25s - 40 s. The measurements reported in this manuscript are recorded with s-SFG, s-Vis, p-mid-IR polarization combination.

For the measurements performed in the region of 1500-1800 cm^{-1}, we use also an amplified Ti:Sapphire laser system (1 kHz, 35 fs, 6.5 mJ/pulse) and generate the SFG signal in the same way as described above. Further, we detect it with a thermoelectrically cooled charged-coupled device (CCD, Princeton Instruments). As already mentioned above, we perform a reference experiment in which the sample is replaced by a z-cut quartz crystal. To obtain high quality data in this frequency region confronts us with several technical challenges. In particular, a so called etaloning effect occurs on the CCD camera. This phenomenon degrades the performance of thinned, back illuminated CCD camera. In order to improve our data quality, we take two independent measurements of z-cut quartz with a different orientation of 180 degrees. By doing so we change the phase of our reference measurement exactly by 180 degrees. If we now add up the two quartz spectra, the interference induced by the SFG signal of the local oscillator and the quartz is removed. The remaining modulation, that largely represents the structural noise of the etaloning effect, can now be used as a scaling factor to remove the structural noise from the data. The typical acquisition time of a single HD-VSFG spectrum here is 600 seconds. In all measurements the height of the reference quartz crystal and the sample are controlled by monitoring the VSFG signal on the camera with a precision of 1 pixel, which, based on the geometry or the setup, results in a phase uncertainty of~π/10 (~20 deg). Also all measurements reported in this frequency region in this manuscript are recorded with s-SFG, s-Vis, p-mid-IR polarization combination.

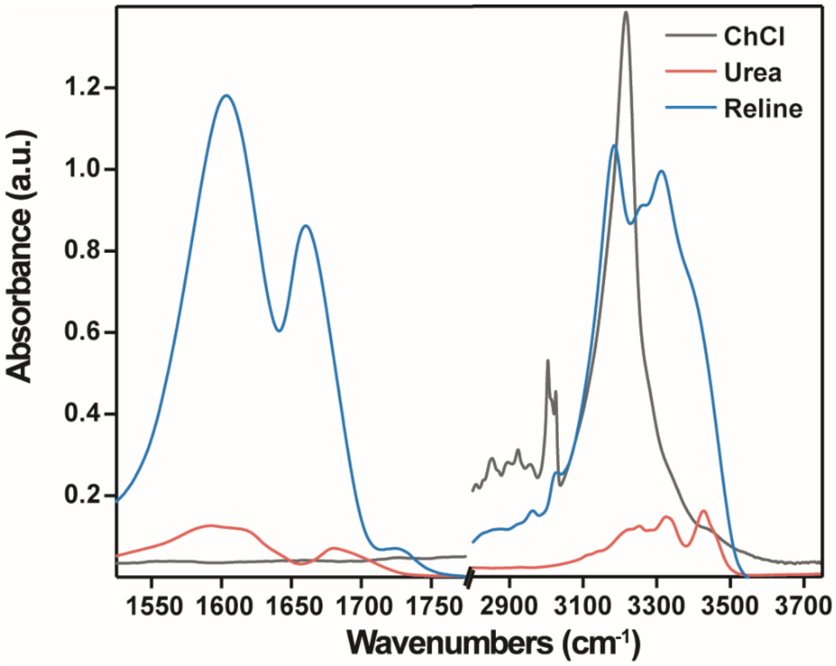

Figure S1. FTIR spectrum ChCl , urea and reline acquired in ATR mode.

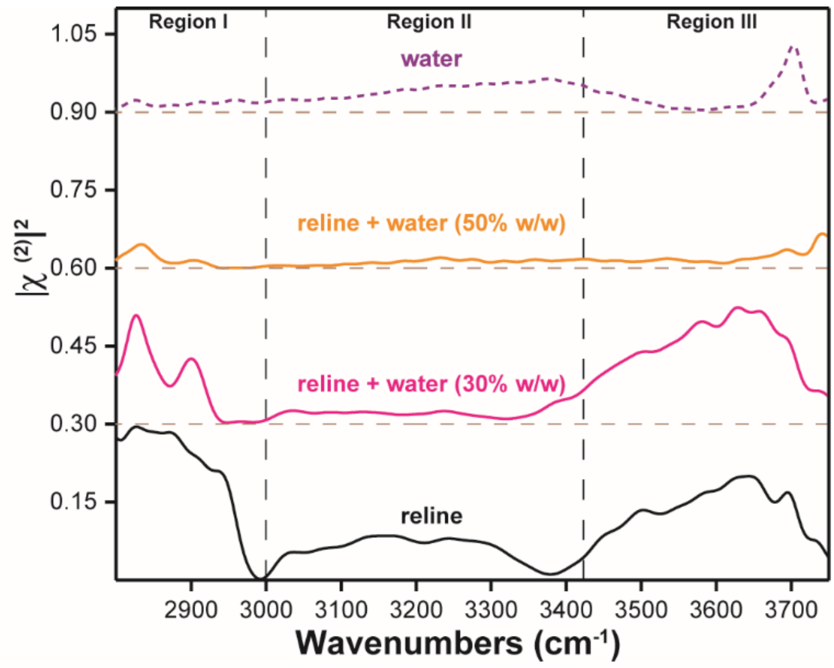

Figure S2. Intensity spectrum of the DES Reline with increasing addition of water by weight percentage from 0% to 50% obtained with HD-VSFG. Region I represent the CH stretch vibrations of ChCl, Region II represent the N-H stretch vibrations from urea and Region III represent the O-H stretch vibrations from ChCl. The spectrum are offset by 0.3 a.u. on the y-axis. The brown dashed line depicts the zero-line for the offset spectrum.

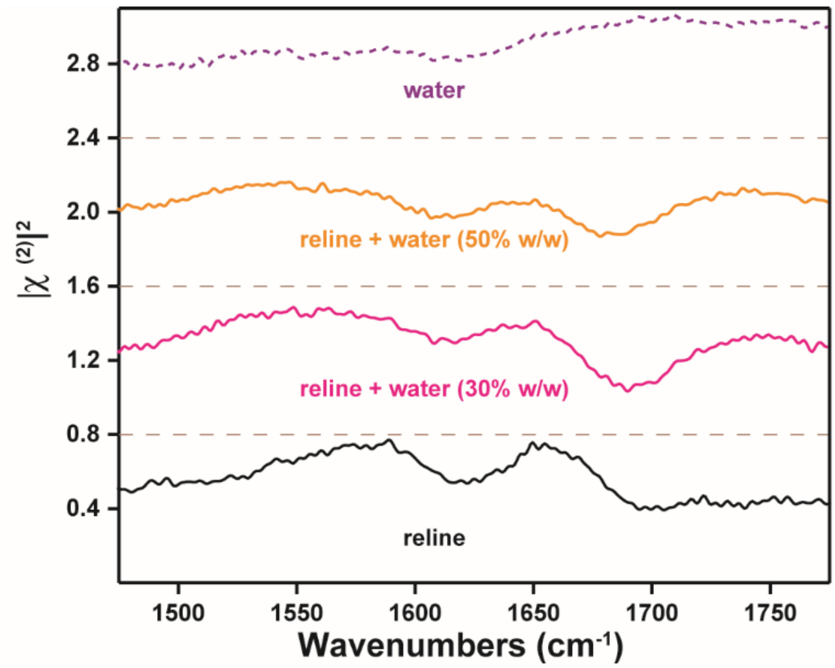

Figure S3. Intensity spectrum of the DES Reline with increasing addition of water by weight percentage from 0% to 50% obtained with HD-VSFG. The spectrum are offset by 0.8 a.u. on the y-axis. The brown dashed line depicts the zero-line for the offset spectrum.

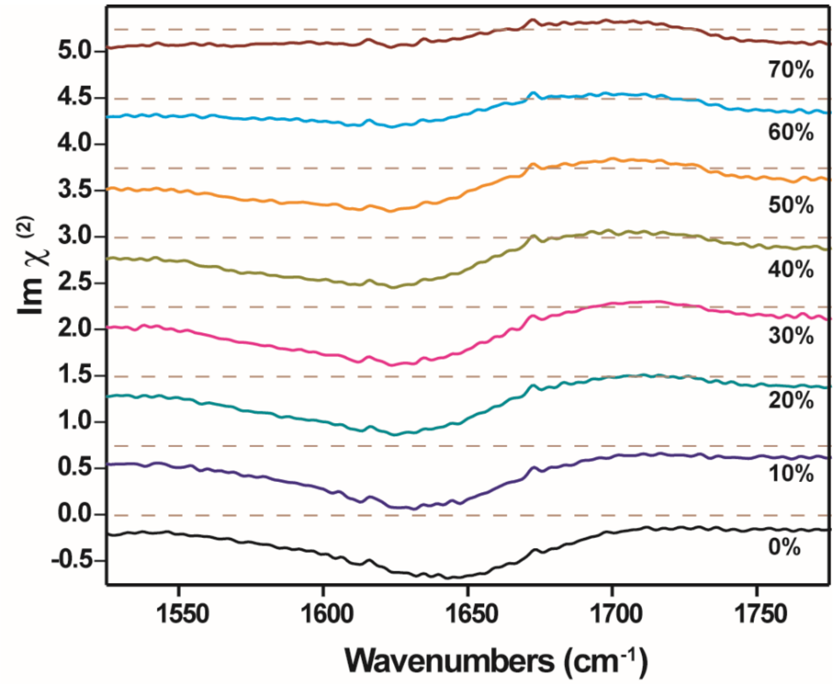

Figure S4. Im$\chi^{(2)}$ spectrum of Reline prepared with d4-Urea and ChCl with addition of D_2O by increasing w/w percentage obtained with HD-VSFG in region of ~1500 - ~1800 cm^{-1}. The spectrum are offset by 0.75 a.u. on the y-axis. The gray line depicts the zero-line for the offset spectrum.

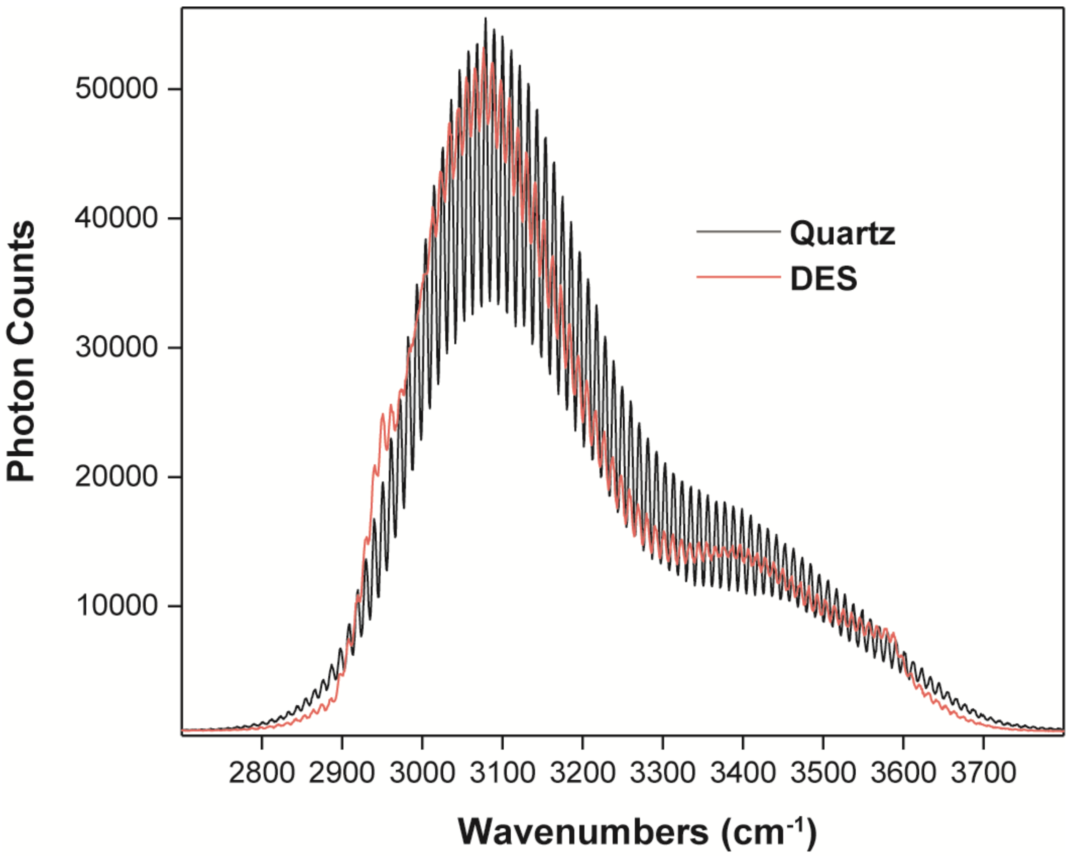

Figure S5. Raw data acquired for the HD-VSFG measurement.

Table S1. Representation of water content used in the study used in w/w% and mol%

**Water weight per cent**	**Number of moles, ChCl:U:W**	**Water mole fraction percent**
**0**	**1:2:0**	**0**
**10**	**1:2:1.6**	**~35**
**20**	**1:2:3.6**	**~55**
**30**	**1:2:6.2**	**~67**
**40**	**1:2:9.6**	**~76**
**50**	**1:2:14.4**	**~83**
**60**	**1:2:21.6**	**~88**
**70**	**1:2:33.6**	**~92**

REFERENCES:

(1) Shen, Y. R. *Fundamentals of Sum-Frequency Spectroscopy*; Cambridge University Press: Cambridge, 2016.

(2) Wang, H.-F.; Gan, W.; Lu, R.; Rao, Y.; Wu, B.-H. Quantitative spectral and orientational analysis in surface sum frequency generation vibrational spectroscopy (SFG-VS) *Int. Rev. Phys. Chem.* **2005**, *24*, 191-256.

(3) Strazdaite, S.; Versluis, J.; Backus, E. H. G.; Bakker, H. J. Enhanced ordering of water at hydrophobic surfaces *The Journal of Chemical Physics* **2014**, *140*, 054711.

(4) Strazdaite, S.; Meister, K.; Bakker, H. J. Orientation of polar molecules near charged protein interfaces *PCCP* **2016**, *18*, 7414-7418.
